# Effects of Machiavellianism on Cyberbullying Perpetration: Serial Mediating Role of Perceived Social Support and Problematic Internet Use Among University Students

**DOI:** 10.11621/pir.2025.0306

**Published:** 2025-09-01

**Authors:** Maryam Javed, Aisha Zubair, Nurmeen Bakhtawar Niazi, Irum Aslam

**Affiliations:** a University of Freiburg, Germany; b National Institute of Psychology, Quaid-i-Azam University, Islamabad, Pakistan

**Keywords:** cyberbullying perpetration, machiavellianism, perceived social support, Problematic Internet Use, internet addiction, university students

## Abstract

**Background:**

Cyberbullying is an increasing concern among university students, notably in Pakistan, where digital engagement is rising. Personality traits like Machiavellianism— characterized by manipulation, callousness, and strategic exploitation— have been implicated in antisocial online behaviors. However, the mechanisms through which Machiavellianism influences cyberbullying remain underexplored.

**Objective:**

To examine the relationship between Machiavellianism and cyberbullying perpetration among university students. Specifically, to explore the serial mediating roles of perceived social support and Problematic Internet Use in this relationship.

**Design:**

The study utilized a purposive sample of 433 university students aged 18 to 25 years (*M* = 21.17, *SD* = 1.89). Participants completed the Machiavellianism Subscale of the Short Dark Triad, the Multidimensional Scale of Perceived Social Support, the Problematic Internet Use Questionnaire, and the Cyberbullying Perpetration Scale to assess the relevant variables.

**Results:**

Machiavellianism and Problematic Internet Use were significant positive predictors of cyberbullying perpetration. Conversely, perceived social support was a significant negative predictor of cyberbullying tendencies. Additionally, both perceived social support and Problematic Internet Use served as serial mediators in the relationship between Machiavellianism and cyberbullying perpetration.

**Conclusion:**

The findings highlight a pathway linking Machiavellianism to cyberbullying through diminished social support and Problematic Internet Use. These results contribute to the understanding of how personality traits can shape online behaviors and offer a framework for future research exploring the psychosocial dynamics of cyberbullying in emerging digital contexts.

## Introduction

The internet has fundamentally transformed social interaction and information exchange worldwide, including among university students. Alongside these benefits, increased online engagement has introduced complex challenges affecting psychological health, social relationships, and personal security (Nakshine et al., 2022). One significant concern is cyberbullying — deliberate, repeated aggression conducted through digital platforms — which threatens mental well-being and academic success globally (Li et al., 2022). The factors influencing cyberbullying are multifaceted, involving both individual personality traits and social environmental elements.

Among personality factors, Machiavellianism — a trait characterized by strategic manipulation, emotional detachment, and cynical attitudes— may increase the likelihood of engaging in cyberbullying (Wright et al., 2022). Moreover, social dynamics such as perceived social support from friends, family, and significant others can shape individuals’ online behavior, potentially mitigating or exacerbating these tendencies. Problematic Internet Use, defined as excessive or poorly controlled online activity, may also act as a behavioral pathway linking personality traits to cyberbullying (Yudes et al., 2022). Despite the growing body of research on cyberbullying, there are few studies on the co-occurrence of Machiavellianism and cyberbullying perpetration, and the factors that can mediate the effect of the former on the latter have not been clarified. This study aims to fill this gap by examining these relationships among university students.

### Machiavellianism as Predictor of Cyberbullying Perpetration

Machiavellianism is an antisocial personality trait characterized by a strategic orientation toward manipulation, long-term planning, cynicism, and a pragmatic disregard for others, often accompanied by low overt aggression (Blotner & Bergold, 2022). Individuals high in Machiavellianism tend to be ambitious, calculating, and adept at controlling social situations through deceptive and emotionally detached behaviors (Miller et al., 2017). Cyberbullying perpetration refers to the intentional and repeated use of digital communication platforms to harass, intimidate, or harm others (Marciano et al., 2020).

The literature upholds the possibility that personality traits play a significant role in predicting cyberbullying perpetration. Regarding personality, the Dark Triad has been identified as a significant precursor to cyberbullying perpetration (Wright et al., 2020). Among these traits, Machiavellianism has consistently emerged as one of the strongest predictors (Safaria et al., 2020). Although psychopathy and narcissism have also been linked to online aggression, Machiavellianism’s unique blend of strategic manipulation, emotional detachment, and instrumental goal-seeking may make it particularly relevant in the context of cyberbullying, where anonymity and reduced social accountability prevail.

Numerous studies (Brown et al., 2019; Panatik et al., 2022; Wagner & Yu, 2021; Wright et al., 2022; Yuan et al., 2020) have demonstrated a significant association between Machiavellianism and tendencies toward cyberbullying. Machiavellianism contributes to cyberbullying perpetration through several key psychological mechanisms. It prompts cyberbullying through a few key variables, including emotion dysregulation (Schade et al., 2021), callous-unemotional traits (Fang et al., 2022), diminished empathy (Yuan et al., 2020), fear of missing out (Servidio et al., 2021), and moral disengagement (Gajda et al., 2022). Alongside being deceitful, manipulative, and exploitative by nature, Machiavellian individuals also fear social dismissal (Demircioglu & Isil, 2020) and favor the apparent well-being of the online realm, which they can manipulate easily. Cyberbullying provides perpetrators with a feeling of power when they feel defenseless in their real-life endeavors (Kircaburun et al., 2018). Jablonska and Zajdel (2020) found that Machiavellian personalities use the internet to achieve their own agendas, feel safe and unpunished there, want to hack a website, prefer a false online identity, and feel powerful there. Based on empirical derivations, the following hypothesis is posed:

H1: *Machiavellianism positively predicts cyberbullying perpetration.*

### Mediating Role of Perceived Social Support

Perceived social support (PSS) is often understood as support received from external sources; however, a growing body of literature emphasizes its subjective nature and its roots in internal psychological factors, particularly personality traits. Research supports the idea that PSS is more trait-like than state-like and can be influenced by an individual’s disposition (Kitamura et al., 2002; Sarason et al., 1986). Perceived support reflects not just the availability of help but an individual’s belief in that availability, often shaped by their emotional and interpersonal dispositions (Barrera, 1986; Eagle et al., 2019).

Attachment style plays a crucial role in this dynamic. Research has shown that secure attachment is associated with higher levels of PSS (McLeod et al., 2020). Drawing on Life History Theory (Belsky et al., 1991), individuals with stable and nurturing early relationships typically associated with secure attachment, are more inclined to perceive their social environments positively. In contrast, those with unstable attachments, such as individuals high in Machiavellianism, tend to perceive their relationships as unreliable or manipulative (Abell et al., 2014; Tajmirriyahi et al., 2021). Studies have shown that individuals high in Machiavellianism report poor-quality relationships with family (Lang & Birkas, 2014), increased sibling conflict and emotional distance (Ferencz et al., 2022), lower trust and closeness in friendships (Abell et al., 2016). These dysfunctional relational patterns likely carry over into broader social networks, resulting in a lower sense of perceived support. However, individuals with prosocial traits generally report higher levels of PSS (Xu et al., 2022), whereas those with antisocial or manipulative tendencies experience impaired relational functioning, which undermines their sense of being supported. Consistent with this, several studies report a significant negative correlation between Machiavellianism and PSS (Borukanlu & Manee, 2023; Kellett & Sarah, 2008).

Studies suggest that those who have experienced a great deal of criticism or ignorance tend to develop negative generalized interpersonal expectations, which makes them more hostile and aggressive (Smith et al., 2004). A longitudinal study found that lower levels of PSS can predict all types of aggressive behaviors one year later (Wright & Wachs, 2019). Cyberbullying can be the “best” option for people with elevated aggressive tendencies, because physical presence is not required. Moreover, maintaining anonymity is optional, which makes the offender free from social pressure and moral hesitation. Hence, a lack of social support may make cyberbullying more likely (Yang et al., 2021). Given this evidence, perceived social support is more theoretically and empirically aligned with the psychological mechanisms underpinning cyberbullying among individuals high in Machiavellianism. Therefore, it is hypothesized that:

H2a. *Perceived social support mediates the relationship between Machiavellianism and cyberbullying perpetration.*

### Mediating Role of Problematic Internet Use

Problematic Internet Use (PIU) is increasingly recognized as a broad construct that encompasses various maladaptive patterns of online behavior. It reflects difficulties in controlling internet use that negatively affect daily functioning, relationships, or mental health (Sakakihara et al., 2019). Davis (2001) made a distinction between two types of pathological internet use: a general type, in which excessive internet use is not focused on a single activity, and a specific type, in which dependence is connected to particular internet functions or applications, such as online gaming or gambling. Many different words have been used to conceptualize this behavior. The terms Internet disorder (Pontes & Griffiths, 2014), Internet dependency (Dowling & Quirk, 2009), pathological internet usage (Davis, 2001), and Internet addiction (Young, 1998) have also been used. This language is questionable because it overestimates a pathological condition based on its own standards of addiction to drugs or gambling while ignoring the wide range of harmful internet behaviors. As a substitute, Problematic Internet Use (PIU) is increasingly being utilized as an umbrella term (Fineberg et al., 2018). Because internet use is viewed as a generic activity, this study also utilized the term Problematic Internet Use to encompass the full spectrum of issues.

Building upon this conceptual framework, the current study explores how PIU functions as an explanatory pathway linking personality traits — particularly Machiavellianism—to negative online behaviors such as cyberbullying. Numerous studies have highlighted the pivotal role of personality traits in contributing to Problematic Internet Use. For instance, online behavior can be influenced by personality traits, as individuals may attempt to compensate for unfulfilled needs in the offlline world. Therefore, a person’s personality should be considered as a determining factor in their online behavior (Kircaburun & Griffiths, 2018). Later, Lee et al. (2021) revealed that dark personality traits exert a noteworthy influence on Problematic Internet Use, even after accounting for the impact of the five core personality traits. Machiavellianism can make it difficult for people to interact in person; instead, they may prefer online communication, and owing to their high emotional dysregulation (Hussain et al., 2021), poor agreeableness and emotional intelligence, as well as fear of missing out, they are likely to experience Problematic Internet Use as a result (Servidio et al., 2021). Problematic Internet Use might stem from pre-existing offlline psychopathologies, easily transferred to the online realm, thereby linking Machiavellianism to Problematic Internet Use (Jablonska & Zajdel, 2020). Numerous studies have explored this positive correlation between Machiavellianism and Problematic Internet Use (Hussain et al., 2021; Servidio et al., 2021).

According to problem behavior theory (Jessor, 1987), maladaptive personality traits increase the likelihood of engaging in problematic behaviors. Individuals high in Machiavellianism may exhibit Problematic Internet Use as a result of their manipulative and emotionally detached tendencies. Furthermore, the theory suggests that involvement in one form of maladaptive behavior heightens the risk of engaging in others (Chu et al., 2021). In this context, Problematic Internet Use might catalyze cyber bullying perpetration. Thus, Problematic Internet Use may be strongly associated with a higher likelihood of cyberbullying perpetration (Yudes et al., 2022). Moreover, cyberbullying was linked to Problematic Internet Use as well as behaviors including gambling, sexting, and contacting strangers (Sanmartin et al., 2021). In addition, a growing body of research has shown that excessive internet use can have a negative influence on risk-taking behaviors, interpersonal relationships, mental health, and academic performance (Arrivillaga et al., 2020; Kokka et al., 2021; Teng et al., 2020). It eventually leads to academic failure, damage to family and peer relationships, insomnia, depression, suicidal ideation, and substance use (Arrivillaga et al., 2020; Restrepo et al., 2020; Tóth-Király et al., 2021). As a result, those who are addicted to the internet display higher levels of hostility (Dhaka & Naris, 2019) and may act more violently (Agbaria, 2021). Cyberbullying can serve as a maladaptive reaction in this context. Consequently, it is reasonable to assume that Problematic Internet Use may encourage cyberbullying perpetration. To the best of the researcher’ knowledge, however, no empirical study has determined the mediating role of Problematic Internet Use in the relationship between Machiavellianism and cyberbullying perpetration. Based on the above-mentioned literature, the following hypothesis is posed:

H2b. *Problematic Internet Use mediates the relationship between Machiavellianism and cyberbullying perpetration.*

### Serial Mediating Role of Perceived Social Support and Problematic Internet Use

The serial mediating role of perceived social support and Problematic Internet Use between Machiavellianism and cyberbullying perpetration can be explained through the lens of problem behavior theory (Jessor, 1987). The theory postulates that the interaction of personality traits and the perceived environment affects the engagement in problem behaviors. In line with problem behavior theory, an individual’s perception of the environment is shaped by personality traits that can significantly impact their behavior. Individuals with Machiavellian tendencies tend to adopt an unjust and precarious worldview. Their perspective on human nature is notably negative, often perceiving others as untrustworthy and selfish. Consequently, Machiavellianism shows a significant negative correlation with perceived social support (Kellett & Sarah, 2008). For instance, those high in Machiavellian traits typically report lower-quality relationships, marked by conflict and emotional distance, particularly within family contexts (Ferencz et al., 2022).

This perceived lack of social support can have emotional and psychological consequences (Harrison et al., 2022), such as loneliness, unmet relational needs, and feelings of rejection, which individuals may attempt to manage through online engagement (Machado et al., 2023). The theory of compensatory internet use (Karde-felt-Winther, 2014) proposes that individuals use the internet to cope with stressors in their offline lives. When individuals lack adequate emotional support from real-life relationships, they may turn to online spaces as a substitute for connection and validation. The anonymity, accessibility, and responsiveness of online platforms make them an appealing alternative for emotionally unfulfilled individuals. Over time, this compensatory behavior can become excessive, leading to Problematic Internet Use (Uçur & Donmez, 2021). Supporting this, Prievara et al. (2019) found that individuals with low perceived social support are more likely to use the internet in a maladaptive way to fulfill their unmet social needs. The internet, rather than serving as a healthy coping tool, becomes a space of dependency that replaces rather than supplements offline interactions. Therefore, it is not simply the absence of support, but the resulting emotional void and coping attempts that contribute to Problematic Internet Use.

In line with Problem Behavior Theory, engaging in one maladaptive behavior increases the likelihood of engaging in others. Thus, individuals involved in Problematic Internet Use may also be more prone to engage in cyberbullying perpetration as a means of expressing frustration, asserting control, or seeking attention in the digital world.

H2c. *Perceived social support and Problematic Internet Use serially mediate the relationship between Machiavellianism and cyberbullying perpetration.*

### The Present Study

Ample evidence exists indicating significant associations between Machiavellianism and cyberbullying perpetration. Nonetheless, there are few studies on the co-occurrence of Machiavellianism and cyberbullying perpetration, and the factors that can mediate the effect of the former on the latter have not been clarified. In view of this, this study aims to establish the association between Machiavellianism and cyberbullying perpetration and to examine the serial mediating role of perceived social support and Problematic Internet Use among university students in the Pakistani context. We posit that perceived social support, as one mediator, influences Problematic Internet Use, acting as a bridge between Machiavellian traits and subsequent cyberbullying actions. Understanding this complex interplay and proposing serial mediation involving perceived social support and Problematic Internet Use offers a nuanced perspective on the pathways through which Machiavellian tendencies might unfold into cyberbullying perpetration in the digital landscape.

## Methods

### Participants

The sample comprised university students (*N* = 433) who were approached from different public and private universities in Pakistan. The data was collected using the purposive sampling approach and the sample included both boys (*n* = 222) and girls (*n* = 211). The age range of the sample varied between 18 years to 25 years (*M* = 21.17, *SD* = 1.89). Most participants fall into the 18–21-year age bracket (*n* = 254, 58.7%). The collected data revealed distinct patterns in internet consumption among the participants. According to the findings, 16% of the respondents dedicate 1 to 3 hours per day to their online activities. A larger proportion, 35%, reported spending 3 to 6 hours daily on the internet, while 20% allocated 6 to 9 hours to their online engagements. Remarkably, 8% of the participants admitted to spending more than 16 hours online each day.

### Measures

#### Machiavellianism Subscale (Short Dark Triad)

In the current study, the Machiavellianism subscale from the Short Dark Triad Scale (Jones & Paulhus, 2014) was used to assess Machiavellian tendencies. It uses a 4-point Likert scale ranging from 1 (Strongly Disagree) to 4 (Strongly Agree). The scale comprised nine items. In the present study, scores on the Machiavellianism subscale could range from 9 to 36. A high score on the scale indicates a higher tendency toward Machiavellianism, and a low score represents a higher inclination toward Machiavellianism. Previous studies demonstrated that the Machiavellianism subscale has good reliability, with an alpha coefficient of .76 (Jones & Paulhus, 2014). Likewise, in this study, the scale exhibited a noteworthy alpha reliability of .75.

#### Multidimensional Scale of Perceived Social Support

The Multidimensional Scale of Perceived Social Support (Zimet et al., 1988) was used to measure perceived social support from three sources: family, friends, and significant others. It uses a 4-point Likert scale ranging from 1 (Strongly Disagree) to 4 (Strongly Agree). The scale comprised 12 items. In the present study, the score range on the Multidimensional Scale of Perceived Social Support was 12 to 48, with a higher score representing a higher level of perceived social support, and a lower score indicating lower perceived social support. In previous studies, the Multidimensional Scale of Perceived Social Support has exhibited excellent reliability, with Cronbach's alpha of .88 (Zimet et al., 1988). In the present study, the scale also exhibited a noteworthy reliability of .89.

#### Problematic Internet Use Questionnaire

The current study utilized the Problematic Internet Use Questionnaire (Thatcher & Goolam, 2005). It is comprised of 20 items and has three dimensions: online preoccupation, adverse effects, and social interactions. It uses a 4-point Likert scale ranging from 1 (Strongly Disagree) to 4 (Strongly Agree). The total scores ranged from 20 to 80, with higher scores exhibiting a higher level of Problematic Internet Use and lower scores indicating a lower level of Problematic Internet Use. The Problematic Internet Use Questionnaire demonstrated good reliability, with a coefficient of .87 (Thatcher & Goolam, 2005). In the present study, the alpha reliability of this scale was found to be .87.

#### Cyberbullying Perpetration Scale

The scale was originally developed by Lee et al. (2017) and later adapted by Iqbal and Jami (2021). The adapted version of the Cyberbullying Perpetration Scale was used in this study. It was a 4-point Likert Scale, where 1 = Not at all, 2 = Rarely, 3 = Sometimes, and 4 = Often. The total possible score ranges from 27 to 108, with higher scores indicating a higher inclination towards cyberbullying perpetration and lower scores suggesting a lower level of cyberbullying perpetration. The scale demonstrated excellent internal consistency, with a Cronbach’s alpha reliability coefficient of α = .93 (Lee et al., 2017). In the present study, it also exhibited excellent reliability with Cronbach’s alpha coefficient of α = .93.

### Procedure

For data collection, students were approached from different universities. Before providing them with the questionnaire, respondents were briefed about the topic, aims, objectives, and significance of this research. Inclusion criteria involved being currently enrolled in university and willingness to participate voluntarily. Exclusion criteria included adolescents with known or self-reported psychological disorders or traumatic experiences, as such conditions could confound the study variables. Participants were asked to confirm the absence of any formal psychological diagnoses or treatment history during screening. Participants were informed that they had the right to quit at any time during data collection. After the briefing, consent forms, demographic sheets, and scales were given to students for completion, with the assurance that their information would be kept confidential and would be used for this research. A total of 450 questionnaires were distributed, out of which 433 complete and valid responses were received, resulting in a response rate of approximately 94%. The participants were appreciated for their time and cooperation and were thanked for providing genuine information.

Participation was completely voluntary, and no monetary or academic compensation was provided. Participants were assured that their responses would be kept strictly confidential. Data were anonymized by assigning unique identification codes, and no personally identifiable information was collected. All data were securely stored on a password-protected university server, accessible only to the principal investigator and research supervisor. The study adhered to ethical standards outlined by the American Psychological Association (APA) and received ethical approval from the relevant institutional review board prior to data collection.

## Results

The Statistical Package for Social Sciences was used for the study’s initial analysis of its variables. Analysis performed for the results of main study include correlation analysis for the correlation of the variables with each other and the subscales. Regression analysis was performed to check the variability caused by the predictors to the outcomes. Analysis was done to assess the role of perceived social support and Problematic Internet Use as serial mediators between Machiavellianism and cyberbullying perpetration.

### Descriptive Statistics

Mean, standard deviation, Cronbach’s alpha reliability, skewness, and kurtosis along with range of the data were tabulated. Potential range *Table 1* indicates the score range obtained by the sample, while the actual range is the range of the scale between which scores can fall. Skewness and kurtosis were calculated for the normality assumptions. *Table 1* shows that the Cronbach’s alpha reliabilities of all the scales and subscale fall in an acceptable range .67 to .93, which indicates that the scales accurately measure the constructs and are internally consistent. The values of skewness and kurtosis fall between +2 and –2, which indicates that the data is normally distributed and that it may be subjected to parametric tests. Standard deviation of the scales indicates that the variability of the data is normally distributed. The score range of the scales and subscales falls between actual ranges of the scales.

**Table 1:** Descriptive Statistics and Cronbach’s Alpha of the Scales Used in the Current Study (N = 433)

Scale Title	*k*	*α*	*M*	*SD*	Skew	Kurt	Range	
							Potential	Actual
Machiavellianism subscale	9	.75	24.72	3.75	–.40	.27	9–36	12–34
MDSPSS	12	.89	30.95	8.65	–.07	–1.08	12–48	12–48
Family subscale	4	.81	10.58	3.39	–.10	–1.07	4–16	4–15
Friend subscale	4	.77	10.27	3.15	–.07	–.90	4–16	4–13
Significant other subscale	4	.82	10.09	3.47	–.01	–1.10	4–16	4–16
PIUQ	20	.87	42.87	10.83	.03	–.69	20–80	21–69
Online preoccupation	10	.81	22.36	6.14	.01	–.80	10–40	10–39
Adverse effects subscale	7	.75	13.88	4.26	.36	–.68	7–28	7–26
Social Interaction Subscale	3	.67	6.62	2.14	.15	–.53	3–12	3–11
CBPS	26	.91	48.48	15.57	.52	–.81	27–108	27–93
Written/Verbal Subscale	11	.86	19.51	6.75	.59	–.82	11–44	11–40
Visual/Sexual Subscale	5	.77	8.78	3.44	.85	–.11	5–20	5–20
Cyber Exclusion Subscale	4	.69	7.65	2.67	.62	–.14	4–16	4–15
Cyber mobbing Subscale	7	.81	10.75	4.11	.65	–.65	7–28	7–23

*Note. MDSPSS = Multidimensional Scale of Perceived Social Support; PIUQ = Problematic Internet Use Questionnaire; CBPS = Cyberbullying Perpetration Scale; k = No. of items; α= Cronbach’s alpha.*

*Table 2* indicates the correlation pattern among the study variables. The results show that Machiavellianism is significantly positively correlated with Problematic Internet Use and cyberbullying perpetration. Machiavellianism is significantly negatively correlated with perceived social support. Moreover, Problematic Internet Use has a significant positive correlation with cyberbullying perpetration and a significant negative correlation with perceived social support. Perceived social support is significantly negatively correlated with cyberbullying perpetration.

**Table 2:** Pearson Product Moment Correlation Among All Study Variables (N = 433)

S. N.	Var	1	2	3	4	5	6	7	8	9	10	11	12	13	14
1	Mach	–	–.25	–.18	–.22	–.16	–.26	–.22	–.24	–.20	–.32	–.31	–.20	–.27	–.30
2	PSS		–	.84	.87	.86	–.19	–.06	–.32	–.07	–.51	–.49	–.50	–.39	–.48
3	Fam			–	.62	.57	–.17	–.09	–.28	–.09	–.43	–.49	–.41	–.33	–.43
4	Fr				–	.67	–.11	–.06	–.26	–.07	–.49	–.41	–.39	–.33	–.42
5	SO					–	–.17	–.05	–.28	–.09	–.48	–.38	–.42	–.34	–.39
6	PIU						–	.91	.82	.76	.44	.39	.32	.41	.40
7	OP							–	.58	.64	.30	.26	.21	.31	.29
8	AE								–	.56	.44	.39	.41	.37	.42
9	SI									–	.25	.21	.17	.25	.26
10	CBP										–	.89	.84	.76	.90
11	W/V											–	.72	.63	.76
12	V/S												–	.55	.71
13	CE													–	.62
14	CM														–

Note. Mach = Machiavellianism; PIU = Problematic Internet Use; OP = online preoccupation; AE = adverse effects; SI = social interaction; PSS = perceived social support; Fam = family; Fr = friend; SO = significant other; CBP = cyberbullying perpetration; W/V = written/visual; V/S = visual/ sexual; CE = cyber exclusion; CM = cyber mobbing. *p < .05. **p < .01.

### Regression Model Predicting Cyberbullying Perpetration

*Table 3* presents the impact of Machiavellianism, Problematic Internet Use, and perceived social support on cyberbullying perpetration. Hierarchical regression was employed to examine the incremental contribution of each predictor in explaining cyberbullying behavior, allowing for a stepwise evaluation of their unique and combined effects.

**Table 3:** Hierarchical Multiple Regression Analysis Predicting Cyberbullying Perpetration (N = 433)

Variables	*B*	95% CI for B		SE *B*	fl	*R* ^2^	Δ*R*^2^
		*LL*	*UL*				
*Step 1*						.37	.37
Constant	61.92^**^	53.66	70.18	4.20			
Machiavellianism	.68^**^	.42	.93	.12	.20^**^		
PSS	–.96^**^	–1.09	–.82	.07	–.53^**^		
*Step 2*						.47	.10
Constant	45.92^**^	37.49	54.35	4.92			
Machiavellianism	.43^**^	.19	.67	.12	.13^**^		
PSS	–.88^**^	–1.01	–.76	.06	–.49^**^		
PIU	.46^**^	.35	.56	.05	.32^**^		

Note. PSS = Perceived Social Support; PIU = Problematic Internet Use; CI = Confidence Interval; LL = Lower Limit; UL = Upper Limit. **p < .001.

In step 1, Machiavellianism and perceived social support explain a 37% variance in cyberbullying perpetration, indicating that approximately 37% of the variance in the dependent variable can be accounted for by Machiavellianism and perceived social support. In step 2, the value of *R*^2^ revealed that predictor Machiavellianism, perceived social support, and Problematic Internet Use collectively explain 47% variance in predicting cyberbullying perpetration. The value of *R*^2^ (Δ*R*^2^) increased from step 1 to step 2, which means that the addition of a new predictor (Problematic Internet Use) in step 2 explains an additional 10% of the variance in the dependent variable.

Collinearity diagnostics were also performed to assess multicollinearity among the predictors. The Variance Inflation Factor (VIF) values were well below the conventional cutoffof 5, with Machiavellianism = 1.12, Perceived Social Support = 1.06, and Problematic Internet Use = 1.09, indicating no multicollinearity concerns.

Overall, *Table 3* presents significant relationships between the predictors (Machiavellianism, perceived social support, and Problematic Internet Use) and the outcome variable. Findings demonstrated that Machiavellianism and Problematic Internet Use positively predicted tendencies of cyberbullying perpetration, whereas perceived social support negatively predicted cyberbullying perpetration.

### Perceived Social Support and Problematic Internet Use as Serial Mediators Between Machiavellianism and Cyberbullying

*Table 4* shows the mediating effect of perceived social support and Problematic Internet Use for the relationship between Machiavellianism and cyberbullying perpetration. The direct effect suggests that, while controlling for the effect of perceived social support and Problematic Internet Use, Machiavellianism has a significant positive relationship with cyberbullying perpetration.

**Table 4:** Serial Mediating Role of Perceived Social Support and Problematic Internet Use for Predicting Cyberbullying Perpetration from Machiavellianism (N = 433)

Variables	*B*	*β*	*p*	95% CI	
				*LL*	*UL*
Constant	45.92		.00	37.49	54.35
Machiavellianism	.43	.13	.00	.19	.67
Perceived Social Support	–.88	–.49	.00	–1.01	–.76
Problematic Internet Use	.45	.31	.00	.35	.56
Indirect Effects					
Machiavellianism → Perceived Social Support	–.40	–.22	.00	–.57	–.23
Machiavellianism → Problematic Internet Use	.54	.23	.00	.32	.75
Perceived Social Support → PIU	–.15	–.12	.00	–.27	–.04
Machiavellianism → PSS → CBP	.36	.10		.21	.52
Machiavellianism → PIU → CBP	.24	.07		.13	.38
Machiavellianism → PSS → PIU → CBP	.02	.00		.00	.01

*Note. PSS = Perceived Social Support; PIU = Problematic Internet Use; CBP = Cyberbullying Perpetration.*

**Figure 1.**
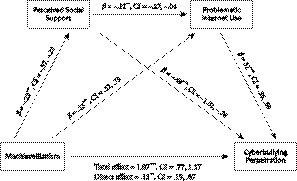
Social support and internet use as mediators between Machiavellianism and cyberbullying (*N* = 433)

*Figure 1* shows the serial mediating effect of perceived social support and Problematic Internet Use for the relationship between Machiavellianism and cyberbullying perpetration. Machiavellianism negatively predicts perceived social support; while perceived social support also negatively predicts Problematic Internet Use. Likewise, Problematic Internet Use positively predicts cyberbullying perpetration. Direct effects and indirect effects all are significant, hence offering empirical support for H2c.

## Discussion

The findings of this study have shown that Machiavellianism positively predicts cyberbullying perpetration among university students in Pakistan. This finding is quite consistent with earlier studies (Panatik et al., 2022; Wagner & Yu, 2021; Wright et al., 2022; Yuan et al., 2020), which also manifested similar patterns to Machiavellianism and cyberbullying perpetration. This finding could be optimally explained through the lens of Problem Behavior Theory (Jessor, 1987), which attempts to explain the link between personality and antisocial tendencies. According to this theory, antisocial personality traits are associated with an elevated chance of participating in problematic activities. The underlying assumption of Problem Behavior Theory is the pivotal role of personal dispositions that may be shaped by the process of socialization and built-in traits and subsequently lead to behavioral tendencies. Given that, morbid personality attributes such as Machiavellianism would have a linear alliance with digital forms of hurting others, such as cyberbullying perpetration (Wright et al., 2022); furthermore, individuals with Machiavellian features tend to achieve their goals as a mechanism of gaining power or control over others and are more likely to indulge in deliberately hurtful behaviors (Miller et al., 2017). Kircaburun et al. (2018) reported that cyberbullying allows perpetrators to gain a feeling of power as they feel powerless in their real-life pursuits. Additionally, individuals high in Machiavellianism may be more likely to use digital technologies as a means of manipulation and deception using social media, for example, to spread rumors or false information about others, to damage their reputation or social standing. Jablonska and Zajdel (2020) asserted that Machiavellian personalities use the internet for achieving their own purposes, feel anonymous and unpunishable online, want to hack a website, prefer a fictional online identity, manipulate other people on the internet, and possess a sense of power online.

Findings further showed the serial mediating effect of perceived social support and Problematic Internet Use in predicting cyberbullying perpetration from Machiavellianism. Perceived social support mediated the relationship between Machiavellianism and cyberbullying perpetration, which is consistent with a previous study (Borukanlu et al., 2023). The indirect effect of perceived social support is elaborated in the social support paradigm (Cullen, 1994), which emphasizes that caring agents inclusive of family, friends, colleagues, and the community at large act as shielding sources to prevent harmful and antisocial outcomes. People with Machiavellian views tended to have a view that the world is unjust and dangerous; they have a negative view of human nature and perceive others as untrustworthy and selfish. Thus, with the increase of one’s Machiavellian views, the level of distrust and fearfulness regarding other human beings also increases (Monaghan et al., 2018). An explanation for this finding could be that Machiavellian individuals may have difficulties in developing genuine and supportive social relationships due to their manipulative tendencies (Abell et al., 2016; Ferencz et al., 2022). This can lead to a perception of low social support, as they may struggle to form authentic connections with others, which is further exacerbated by competitive academic environments, hierarchical teacher-student dynamics, and limited peer emotional support—common features in Pakistani educational culture. These cultural factors may reinforce feelings of isolation and lead such students to seek connection or control through excessive Internet use.

The findings of the present study further revealed that social support can directly influence Problematic Internet Use. This finding is adequately understandable in the context of the theory of compensatory internet use (Kardefelt-Winther, 2014) and validates its applicability in explaining the link between perceived social support and Problematic Internet Use. The theory of compensatory internet use proposes that internet use can be a coping strategy for real-life problems. Individuals lacking social support can use the internet to fulfill their unmet social needs (Prievara et al., 2019), with Problematic Internet Use as a potential consequence (Uçur & Donmez, 2021). One explanation for this relationship is that when individuals feel a lack of social support in their offline lives, they may turn to the internet as a coping mechanism to fulfill their emotional needs. Problematic Internet Use, such as excessive time spent on social media, can provide temporary relief from negative emotions or feelings of loneliness (Prievara et al., 2019). However, this reliance on the internet as a source of comfort can lead to an unhealthy dependence and neglect of real-life relationships. Moreover, low perceived social support can create a sense of dissatisfaction with offline social interactions. As a result, individuals may seek refuge in online communities, where they can escape from their daily problems and experience a sense of belonging. This desire for escapism can drive excessive internet use, as individuals become absorbed in virtual worlds and interactions, neglecting their offline responsibilities and relationships. The internet provides easy access to a wide range of activities and content, making it a convenient outlet for individuals with low perceived social support. Online platforms offer endless entertainment, socialization, and information, which can be appealing to those who feel isolated or disconnected in their offline lives. The constant availability and accessibility of the internet can lead to overuse and neglect of offline relationships and responsibilities.

Furthermore, serial mediation revealed that Problematic Internet Use leads to cyberbullying perpetration. According to problem behavior theory (Jessor, 1987), engaging in one problematic behavior can facilitate the participation of another (Chu et al., 2021). Therefore, Problematic Internet Use may be closely correlated with an increased probability of cyberbullying perpetration (Yudes et al., 2022). Consistent with this viewpoint, empirical studies have reached similar conclusions; for instance, Sanmartin et al. (2021) found that cyberbullying is associated with both Problematic Internet Use and behaviors such as sexting, gambling, and contacting strangers. In the Pakistani context, limited offline support systems and social stigma around emotional expression may leave students, especially those high in Machiavellianism, with fewer avenues to express distress or frustration healthily. Consequently, these students may turn to the internet as a relatively anonymous space for self-expression and release. The sense of anonymity and disinhibition online lowers accountability, emboldening individuals to engage in cyberbullying without fear of immediate consequences. The internet thus provides a hidden platform behind usernames or profiles, facilitating hurtful behaviors while avoiding direct offline repercussions. Additionally, weak institutional monitoring and inadequate mental health support in educational settings may exacerbate these behaviors, underscoring the need for culturally sensitive interventions targeting Problematic Internet Use and cyberbullying.

## Conclusion

The study reveals a complex interplay of psychological factors contributing to cyberbullying perpetration among university students. The findings highlight that individuals with higher levels of Machiavellianism are more likely to engage in cyberbullying, particularly when Problematic Internet Use is present. Conversely, strong social support networks can mitigate these tendencies, underscoring their protective role.

The serial mediation model demonstrates that perceived social support and Problematic Internet Use both play significant roles in the pathway from Machiavellianism to cyberbullying. This suggests that interventions aiming to reduce cyberbullying should focus on both enhancing social support and addressing problematic internet behaviors. By targeting these areas, school counselors, educators, and family therapists can develop more effective strategies to prevent cyberbullying and promote a healthier online environment for young adults. Overall, the study emphasizes the need for comprehensive, multifaceted approaches to tackle the issue of cyberbullying in university settings.

## Limitations and Suggestions

The present study has a few potential limitations that may offer caution while considering the findings. Firstly, the primary focus of the study is on university students which may limit the generalizability of its findings to various age cohorts. Therefore, it would be more appropriate to undertake the investigation of the current paradigm across diverse age groups to determine the role of temporal transitions in relation to the psychosocial development of individuals. Secondly, lack of social dynamics has not been considered in the current study, which may offer a limited view to determine the role of personal dispositions in cyberbullying tendencies. Hence, it is equally important to capture the essence of broader social constructs such as parenting practices, peer relations, and schooling experiences that may shape the inclinations of cyberbullying among young adults. Thirdly, future studies would consider the comprehensive ecological model catering to the ethnic, racial, and linguistic needs of the youth that may make them susceptible to disruptive internet usage and digital forms of bullying. Finally, the study’s cross-sectional design precludes the establishment of causal relationships, warranting the need for longitudinal and experimental investigations to explore the temporal dynamics and developmental trajectories of Machiavellianism, Problematic Internet Use, and cyberbullying perpetration, thereby providing stronger evidence for causal associations.
